# Poster Session I - A85 SOCIAL DETERMINANTS OF HEALTH AND HEALTH OUTCOMES IN CELIAC DISEASE: A SCOPING REVIEW

**DOI:** 10.1093/jcag/gwaf042.085

**Published:** 2026-02-13

**Authors:** C Nie, E Rival, S Kassel, M Fields, A Puran, J Cunningham, C M Walsh

**Affiliations:** University of Toronto Temerty Faculty of Medicine, Toronto, ON, Canada; University of Toronto Temerty Faculty of Medicine, Toronto, ON, Canada; University of Toronto Temerty Faculty of Medicine, Toronto, ON, Canada; McMaster University Faculty of Health Sciences, Hamilton, ON, Canada; The Hospital for Sick Children Child Health Evaluative Sciences, Toronto, ON, Canada; The Hospital for Sick Children, Toronto, ON, Canada; The Hospital for Sick Children Child Health Evaluative Sciences, Toronto, ON, Canada

## Abstract

**Background:**

Celiac Disease (CeD) is a common chronic disease that requires strict life-long adherence to a gluten-free diet (GFD), imposing substantial financial, social, and practical burdens on patients and their families. Adverse social determinants of health (SDoH), such as limited income, education or social support, are known to worsen outcomes in many chronic diseases, yet their impact in CeD remains poorly understood. Investigating how SDoH influence dietary adherence, symptom management, and quality of life could reveal critical barriers to care and inform targeted interventions to improve patient outcomes.

**Aims:**

We aimed to synthesize the current literature on the relationship between SDoH and health outcomes in patients with CeD and identify gaps for further research.

**Methods:**

A scoping review was conducted following the Arksey and O’Malley framework and the PRISMA-ScR checklist. Studies published from January 2000 to April 2025 were identified from MEDLINE, Embase, PsycInfo, CINAHL, and SCOPUS databases. Eligible studies reported on both clinical or patient reported outcome measures and SDoH, defined according to the five domains outlined in Healthy People 2030. Two independent reviewers screened titles, abstracts and full-texts, and extracted data on SDoH variables, CeD outcomes, measurement tools, and results.

**Results:**

A total of 2851 articles were screened, of which 66 studies met inclusion criteria. Studies originated from 24 countries, with the most from the US (n = 9), Italy (n = 8) and the UK (n = 6). Among the included articles, 51.5% analyzed the role of SDoH on CeD outcomes as their primary aim, while 48.5% included SDoH in sub-analysis. All five Healthy People 2030 SDoH domains were represented: Economic Stability (54.5%), Education Access and Quality (59.1%), Healthcare Access and Quality (4.5%), Neighborhood and Built Environment (15.2%), and Social and Community Context (84.8%). Reported outcomes were heterogenous with variable methodologies. The most frequently studied CeD outcomes were GFD adherence and health related quality of life (HRQoL). Many studies found that female gender and lower education level were associated with worse HRQoL, although some reported opposite or non-significant associations. All studies examining food insecurity reported a negative association with GFD adherence. Overall, most SDoH variables showed inconsistent associations with CeD health outcomes.

**Conclusions:**

Studies included in this scoping review were of variable quality and reported mixed findings. Few studies examined the domains of Healthcare Access and Quality or Neighborhood and Built Environment. These gaps highlight the need for further focused research to better define the role of SDoH in CeD. Understanding how SDoH influence CeD outcomes is essential to address health disparities and design targeted interventions.

**Funding Agencies:**

None

